# Untangling the Diverse and Redundant Mechanisms of Staphylococcus aureus Biofilm Formation

**DOI:** 10.1371/journal.ppat.1005671

**Published:** 2016-07-21

**Authors:** Marta Zapotoczna, Eoghan O’Neill, James P. O'Gara

**Affiliations:** 1 Department of Clinical Microbiology, Education and Research Centre, Beaumont Hospital, Royal College of Surgeons in Ireland, Dublin, Ireland; 2 Department of Microbiology, Connolly Hospital, Dublin, Ireland; 3 Department of Microbiology, School of Natural Sciences, National University of Ireland, Galway, Ireland; The University of North Carolina at Chapel Hill, UNITED STATES

A major challenge in the management of device-related infections (DRIs) involving microbial biofilms derives from the rapid coating of implanted biomaterials by host-derived glycoproteins and other macromolecules. The performance of modified biomaterial surfaces that limit bacterial colonisation under laboratory conditions is difficult to predict in this in vivo milieu. Biofilms formed by staphylococci have for many decades been recognised as the most frequent cause of biofilm-associated infections with *Staphylococcus epidermidis* and *Staphylococcus aureus* being the two main species of staphylococci associated with DRI. Advances in our understanding of staphylococcal biofilm mechanisms have made one fact clear: namely, that this important pathogen has adopted the mantra "to stick to surfaces at all costs" and employs a remarkable array of adherence mechanisms to achieve this goal. Here we will review these diverse biofilm mechanisms, raise questions about why such redundancy exists, and outline potential implications for the development of new biofilm-targeted therapeutics.

## What Are the Mechanisms of Biofilm Used by Staphylococci?

**Fig 1:**
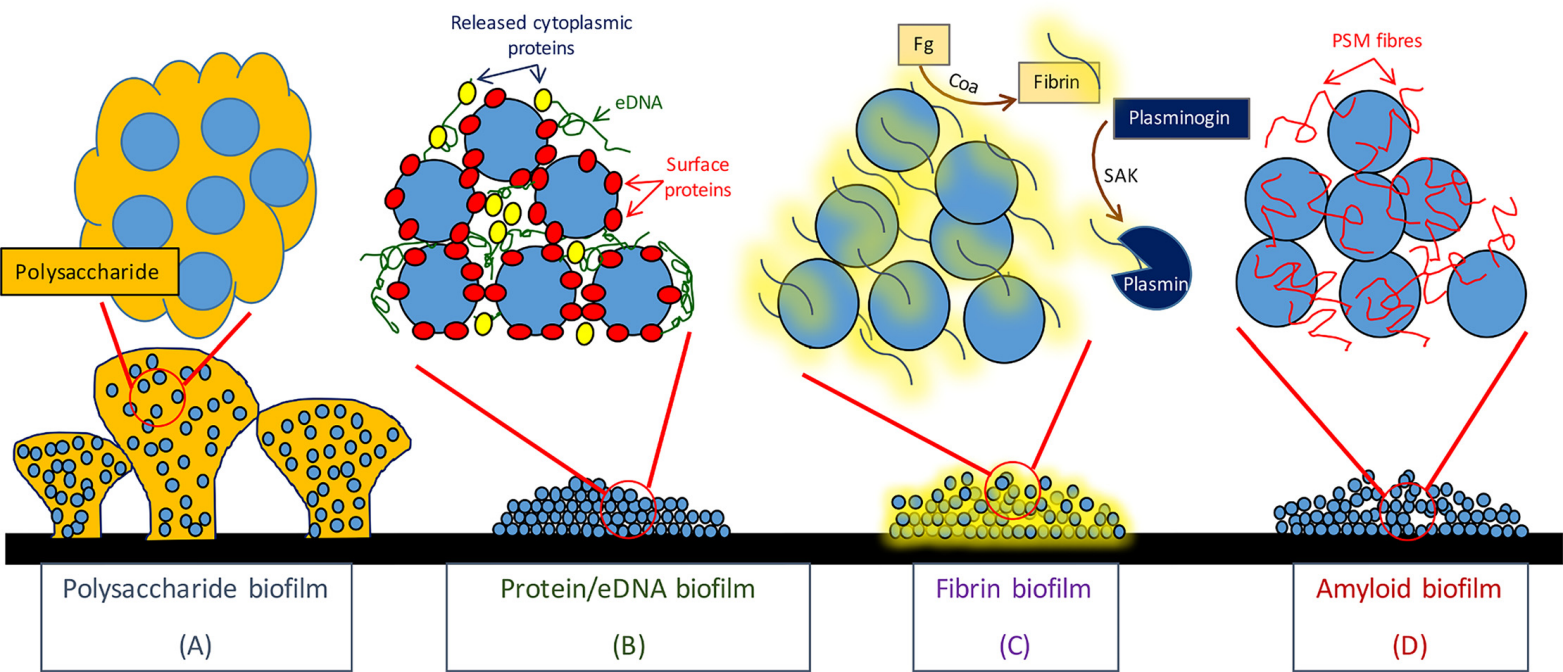
Major mechanisms of biofilm expressed by *S*. *aureus*. (A) Polysaccharide-type biofilm is dependent of expression of PIA/PNAG by intracellular adhesion operon (*icaADBC*)-carrying strains and is common in methicillin-sensitive *S*. *aureus* isolates. (B) Surface proteins such as BAP, the FnBPs, and SasG/Aap mediate direct cell-to-cell contact during biofilm accumulation. Cytoplasmic proteins and eDNA released following cell lysis can also act as components of the biofilm matrix. (C) Coagulase-mediated conversion of fibrinogen (Fg) into fibrin, which is recruited into a biofilm scaffold that can be dispersed by plasmin produced following staphylokinase-mediated activation of plasminogen. (D) Phenol-soluble modules have surfactant qualities that can promote biofilm dispersal but can also accumulate as amyloid aggregates that promote biofilm accumulation.

From the earliest identification of poly-N-acetylglucosamine (PNAG)/polysaccharide intercellular adhesin (PIA) as a first known mediator of *Staphylococcus epidermidis* biofilm formation (reviewed in [1]), interest in this important virulence determinant has led to the discovery of multiple biofilm mechanisms in *S*. *epidermidis* and *S*. *aureus*. The LPXTG-cell wall-anchored biofilm-associated protein (BAP) in bovine mastitis *S*. *aureus* isolates [2], the accumulation-associated protein (Aap) in *S*. *epidermidis* [3], and the fibronectin binding proteins (FnBPs) in human methicillin-resistant *S*. *aureus* (MRSA) isolates [4] were among the first PIA/PNAG-independent biofilm mechanisms to be described. Other protein adhesins include the cell wall-anchored clumping factor A (ClfA), cell wall-anchored clumping factor B (ClfB), *S*. *aureus* surface protein G (SasG), *S*. *aureus* surface protein C (SasC), *Staphylococcus aureus* protein A (Spa), and *S*. *epidermidis* surface protein C (SesC), as well as the cell surface extracellular matrix binding protein (Embp) and extracellular adherence protein (Eap) (reviewed in [5]). Release of extracellular DNA following lysis mediated by the major autolysin contributes to biofilm development in both species [6,7]. Lysis-dependent release of cytoplasmic proteins has also been implicated in the biofilm phenotype [8]. Protein adhesins and extracellular DNA (eDNA) are in turn susceptible to protease and nuclease degradation, which can modulate biofilm development, architecture, and release [9]. The small-peptide toxins, termed the phenol-soluble modulins (PSMs), have surfactant qualities that regulate biofilm maturation and dissemination [10]. PSMs can also aggregate into amyloid structures that enhance biofilm formation [11], building on previously described roles for extracellular amyloid fibres in biofilm formation in other bacteria [11]. Surface charge influenced by wall teichoic acid composition also impacts staphylococcal cell interactions with surfaces and the initiation of biofilm formation [12]. Clearly staphylococci possess an array of biofilm mechanisms (Fig 1), and significant progress has been made over the last number of years in our understanding of the complexity of the various stages involved in staphylococcal biofilm attachment, formation, regulation, and disassembly. Application of this knowledge base and future studies will investigate how interactions between different adhesins influence the biofilm phenotype and the pathogenesis of biofilm-associated infections. Such interactions remain largely unexplored, but studies in a number of bacteria have shown interactions between eDNA and other matrix components such as polysaccharide and amyloid [13–15]. In *S*. *aureus*, interactions between extracellular DNA, amyloid fibres [16], and beta toxin [17] or between PIA/PNAG and teichoic acids [18] have also been reported.

## How Does *S*. *aureus* Exploit the Host Machinery to Build a “Biofilm Shield”?

The ability of *S*. *aureus* to survive in human blood is facilitated by production of coagulase (Coa), which is up-regulated in vivo by the two-component system SaeRS. In the clinical laboratory, Coa or staphylocoagulase production is routinely used to differentiate between *S*. *aureus* isolates and the coagulase-negative staphylococci. Whereas the contribution of surface proteins, secreted and lysis-derived proteins, polysaccharide, and eDNA adhesins, is influenced by strain background, the production of Coa, which we recently reported plays a critical role in biofilm formation under physiologically relevant conditions, is universal for all *S*. *aureus* strains. Upon maturation, like other biofilm types, the fibrin-shielded biofilms exhibit increased resistance to antimicrobial drugs.

Coa binds to host prothrombin forming active staphylothrombin complexes that convert soluble monomeric fibrinogen (Fg) into self-polymerizing insoluble fibrin, which is then recruited by *S*. *aureus* to form the biofilm scaffold [19]. A similar mechanism of fibrin scaffold production is attributed to von Willebrand factor-binding protein (vWbp), a second Coa expressed by *S*. *aureus*. Upon maturation, like other biofilm types, the fibrin-shielded biofilms exhibit increased resistance to antimicrobial drugs [20]. Coa-mediated biofilm formation is clearly dependent on the bacterial cell making contact with Fg as evidenced by the significant role of the Fg-binding surface protein ClfA in this phenotype under high shear [20]. Other microbial surface components recognizing matrix molecules are likely to contribute to adhesion in static or low shear environments. The physiological importance of fibrin-promoted staphylococcal accumulation is further evidenced in abscess formation [21], joint infections, in which antibiotic-resistant, fibrin-embedded bacterial agglomerations in human synovial fluid are a major virulence determinant [22], and in *S*. *aureus* catheter-related infections, which are dependent on the production of fibrin by Coa or vWbp [23]. These studies support a growing body of literature revealing the importance of the fibrin shield protecting *S*. *aureus* from uptake by phagocytic cells and survival in the infection milieu [19,24–26].

## Why Does *S*. *aureus* Retain the Capacity to Express Multiple Apparently Redundant Biofilm Mechanisms?

Given that *S*. *aureus* is highly unlikely to have retained the capacity to express multiple biofilm phenotypes when just one would suffice, it seems reasonable to suggest that these environmentally regulated biofilm mechanisms are niche-specific and may play overlapping roles in both colonisation and biofilm formation. On the skin where NaCl concentrations are relatively high and water availability is low, production of PIA/PNAG may serve primarily to trap water with its role in intercellular adherence a secondary function. Similarly, up-regulation of FnBP expression in host niches where the pH is more acidic (e.g., urinary tract, vagina, mouth, and skin) appear to favour a biofilm mechanism that also promotes bacterial adherence to extracellular matrix proteins such as fibronectin, Fg, and elastin. Indeed, this general hypothesis may also extend to all surface proteins as well as the autolysin-mediated release of cytoplasmic proteins [8] and extracellular DNA with adherence properties [7]. Physiological levels of Zn^2+^, which can be elevated at infection sites, play an important role in promoting Aap/SasG-dependent intercellular adhesion, perhaps in part by altering the cell surface via interactions with negatively charged teichoic acids [27]. On the other hand, the regulation of proteinaceous biofilms by bacterial and host proteases may reflect both a bacterial dispersal mechanism and a host response to infections involving protein adhesin-mediated biofilms.

Under iron- and nutrient-limiting conditions, which are likely to be encountered in blood, the Coa-mediated conversion of Fg to fibrin on surfaces conditioned with plasma proteins promotes a biofilm phenotype that would not be possible outside the host. Limited iron availability in vivo also promotes expression of Embp, Eap, and PIA, which are regulated by the iron regulator Fur and the SaeRS-two component system [28]. Importantly, the SaeRS system controls Coa expression and is required for fibrin-mediated biofilm on plasma coated surfaces [20].

The physiological relevance of the Coa-mediated biofilm suggests that it is likely to play an important role in *S*. *aureus* DRI. However, *S*. *epidermidis* and other Coa-negative staphylococci are also a major cause of DRI despite being genetically incapable of producing fibrin-dependent biofilms and rather use polysaccharide and protein adhesin biofilm mechanisms as described above. Returning to the idea that different biofilm adhesins may play overlapping roles in both colonisation and biofilm formation, it would seem likely that Coa-mediated production of fibrin biofilms may be exploited by *S*. *aureus* for rapid colonisation of implanted devices but that over longer time periods, other biofilm adhesins may play increasingly important roles in the maturation of the biofilm. Supporting this idea, we recently reported that 24-hour fibrin biofilms were significantly more susceptible to inactivation by antibiotics than FnBP-dependent biofilms of the same age but that over time, the fibrin biofilms became increasingly resistant [20].

## What Are the Implications of These Different Biofilm Mechanisms for Future Therapeutics?

As noted above, comparative studies revealed that the antibiotics daptomycin, tigecycline, and rifampicin were capable of an almost complete inactivation of 24-hour fibrin-mediated biofilms, whereas FnBP-mediated biofilms were significantly more resistant [20]. A recent study using an antibiotic lock model of infection showed that very high doses of these antibiotics retained significant activity against mature three- and five-day biofilms, which is more likely to reflect the “real-life” clinical scenario in which treatment is started following diagnosis of a DRI [29]. Early diagnosis and intervention against biofilm-associated infections may therefore be of significant therapeutic importance using existing antimicrobial drugs, although the need to administer antimicrobials at many thousand times the minimum inhibitory concentration of the organism to achieve adequate biofilm inactivation remains the major challenge.

Dispersal of biofilms by dispersin B, proteases, nucleases, or agents capable of manipulating PSM production have been proposed to have therapeutic potential [9,30]. The isoquinoline alkaloid berberine has been reported to prevent PSM accumulation into amyloid fibrils [31]. Similarly, the fibrin-degrading pathway of the host coagulation cascade, in which activation of host plasminogen generates plasmin, can also be exploited to manipulate *S*. *aureus* biofilm, offering a new therapeutic option to treat *S*. *aureus* DRI. Fibrin-mediated biofilms can be eradicated by plasmin and other fibrinolytic enzymes such as nattokinase or serrapeptase [20]. Kwiecisnki et al. demonstrated that increasing levels of staphylokinase, which activates plasminogen, inhibited biofilm in a mouse catheter infection model [32], whereas pre-coating catheter surfaces with tissue plasminogen activator also inhibited adhesion and biomass accumulation in the same in vivo model [33]. The drug dabigatran (a pharmacological inhibitor of both staphylothrombin and thrombin) inhibited fibrin-mediated biofilm by blocking the interaction between Coa/vWbp and prothrombin both in vitro and in a murine central venous catheter model [23]. In prosthetic joint infections, pharmacological manipulation of the PSM-controlled interaction between PIA/PNAG and the cell surface, which contributes to the agglomeration of fibrin-dependent, biofilm-like cell clusters, has therapeutic potential [22]. Depending on the complexity of biofilms formed in vivo, a combination of biofilm dispersal agents may be necessary. However, because dispersal agents may seed bacteria to other organs, such therapies would need to be used in combination with systemic antimicrobial drugs. Nevertheless, degradation of the biofilm matrix represents a promising therapeutic approach both for prevention and eradication of biofilms. Collectively, these data underscore the importance of future studies to determine which biofilm mechanism (or combination of mechanisms) is deployed by staphylococci in human DRIs and the susceptibility of in vivo-formed biofilms to antimicrobial therapy.

The important role for fibrin shields in staphylococcal virulence, generally [19,24–26], and biofilm-associated infections, specifically, opens up the possibility that active and passive immunization strategies targeting Coa may also represent an effective anti-biofilm strategy. A recent study has shown that monoclonal antibodies raised against the Fg-binding R domain at the C-terminus of Coa marked the fibrin shield for phagocytic killing, protected mice from MRSA sepsis, and enhanced opsonophagocytic killing of MRSA in blood samples from healthy human volunteers [19]. PNAG also shows considerable potential as a vaccine candidate (reviewed in [34]), and a combination vaccine targeting Coa, PNAG, and other biofilm adhesins may also help prevent DRIs. Continued progress in understanding the mechanisms of staphylococcal biofilm and their relevance in different host niches is needed to augment and expand current antimicrobial treatment for these significant infections.
